# Adult with concurrent mesenteric cyst and acute appendicitis: a case report

**DOI:** 10.1093/jscr/rjad275

**Published:** 2023-05-20

**Authors:** Barbara Y Hernandez Cervantes, Duniesky Martinez Lopez, Fernando M Almaguer Acevedo, Manuel Betamcourt Benjamin, Radisnay Guzman Lambert, Mariuska Rodriguez Gonzalez

**Affiliations:** Department of Surgery, School of Medicine, University of Health and Allied Sciences, Ho, Ghana; Department of Internal Medicine, School of Medicine, University of Health and Allied Sciences, Ho, Ghana; Department of Surgery, School of Medicine, University of Health and Allied Sciences, Ho, Ghana; Department of Radiology, School of Medicine, University of Health and Allied Sciences, Ho, Ghana; Department of Surgery, School of Medicine, University of Health and Allied Sciences, Ho, Ghana; Department of Internal Medicine, School of Medicine, University of Health and Allied Sciences, Ho, Ghana

**Keywords:** mesenteric cyst, acute appendicitis, acute appendicitis and CT scan, mesenteric cyst associations

## Abstract

Mesenteric cysts are uncommon intra-abdominal lesions that account for only one in every 100 000 adult hospitalizations. Their diagnosis is based on a comprehensive clinical examination as well as radiological modalities such as ultrasonography and computed tomography (CT) scans, and it is usually a clinical challenge because of non-specific symptoms. We present our first case of a 51-year-old man with a simple mesenteric cyst accompanying acute appendicitis diagnosed by CT scan of the abdomen and treated by exploratory laparotomy, complete enucleation of the cyst and appendectomy with a 10 month follow-up without complications or recurrence. This type of presentation has not been thoroughly investigated, with only two children reported during our literature review. Even if there is a high level of suspicion, a CT scan is required for confirmation.

## INTRODUCTION

Mesenteric cyst is a rare cystic disease that affects one out of every 100 000–250 000 hospitalized patients [1–3]. Beneviene described it first in 1507, and Tillaux was the first to remove the cyst in 1880 [4]. Mesenteric cysts are distinct in that, despite being classified as a separate entity, the clinical presentation, aetiology, radiological features and pathological characteristics vary greatly [1]. Most mesenteric cysts are asymptomatic and are discovered by chance during laparotomies for other pathologies [5]. The clinical examination findings are determined by the size of the cyst. The diagnosis is based on a thorough clinical examination as well as radiological modalities such as ultrasonography and computed tomography (CT) scans [2, 3, 5]. Our patient presented with an unusual combination of a mesentery cyst and acute appendicitis; appendectomy and enucleation of the cyst were done via laparotomy; this co-occurrence was found only in two children during the literature review.

## CASE PRESENTATION

A 51-year-old black male patient presented to our outpatient clinic with a 2-year history of intermittent abdominal pain. The patient was in his regular state of health until 2 years ago, when he began suffering discomfort in the left lumbar region, which then spread to the right lumbar region and right iliac fossa; nonetheless, the pain in the left lumbar region remained persistent. The pain developed gradually, was piercing and did not spread to other portions of his body. Some days he gave it an 8/10 grade, and other times a 4/10. It had no alleviating elements; however, straining during defecation exacerbated it. The pain was accompanied by a lack of appetite, fatigue, headaches and overall body weakness. It was also connected to a painful protrusion that manifested when he lay down. He was treated in a peripheral facility until ~10 months ago, when he was referred to our clinic for further management as his illness worsened and an ultrasound revealed an intra-abdominal mass. On examination, there was no palpable abdominal mass, but the abdomen was painful in the left lumbar region and right iliac fossa, with the pain being most intense at McBurney’s point. There was no rebound tenderness found. Past medical history of inguinal hernia was repaired 6 years ago.

**Figure 1:**
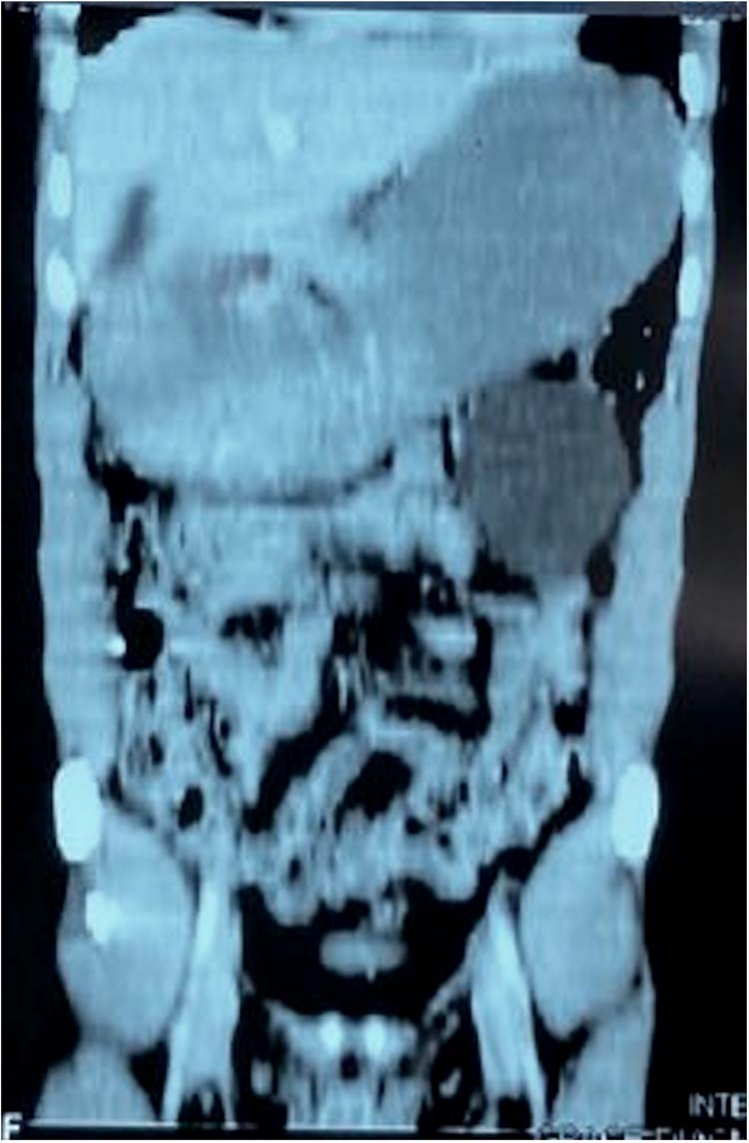
Coronal CT image of the inflamed appendix and mesenteric cyst.

**Figure 2:**
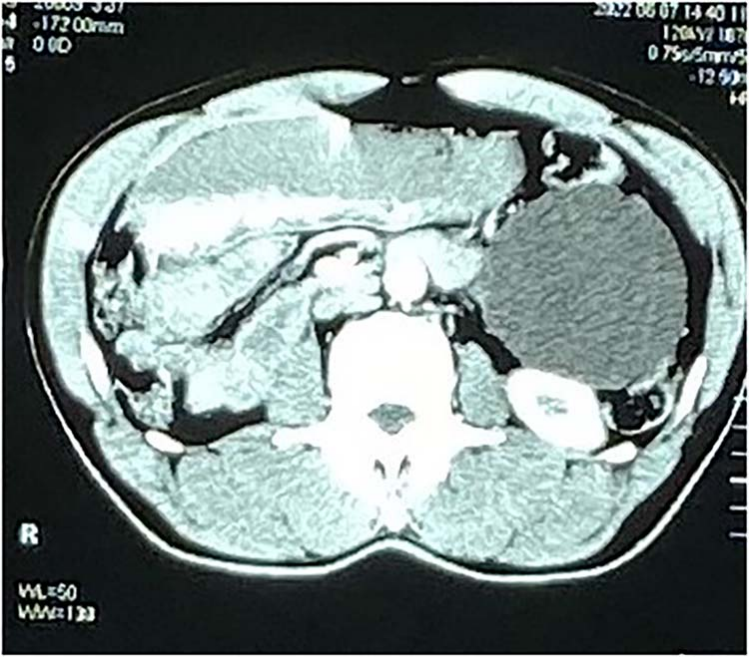
Axial CT image of the mesenteric cyst.

Routine laboratory investigations were done with the following values: HB 14 g/dl, WBC 3.4 × 103 u/l, neutrophils 30.8%, lymphocytes 53%, monocytes 11.2%, eosinophiles 4.5%, and renal and liver function tests were normal. Electrolytes were normal, and erythrosedimentation was 9 mm/h. The abdomen-pelvic ultrasound showed a huge, well-defined, thin-walled cyst with internal debris abutting the anterior-inferior surface of the left kidney, measuring 10 × 7.3 cm. The Doppler interrogation was unremarkable. The caecal appendix was not mentioned, and the suspected diagnosis was a mesenteric cyst. A CT abdominopelvic scan showed a well-defined ovoid intrabdominal mass (9.6 × 8.6 × 8.2 cm) with barely perceptible walls in the left lumbar region (inferior and anterior to the left kidney and lateral to the left psoas muscle). There were no solid-enhancing components or calcifications. It had a minimal compressive effect on the adjacent loops of bowel; also, appendiceal dilatation and wall thickening were noted (Figs 1 and 2), with no signs of mesenteric lymph nodes, appendicoliths or peri-intestinal fluid. There was no evidence of associated ascites or paraaortic lymphadenopathy. It was concluded to be a left lumbar/flanking mesentery cyst and possible appendicitis.

**Figure 3:**
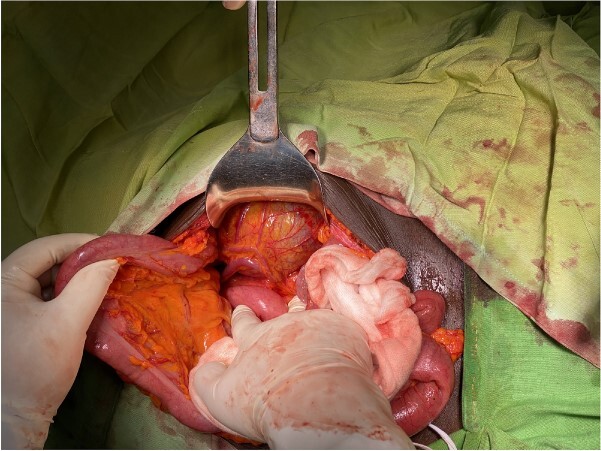
Intraoperative mesenteric cyst located in the descendent mesocolon with left colic vessels overrunning it.

**Figure 4:**
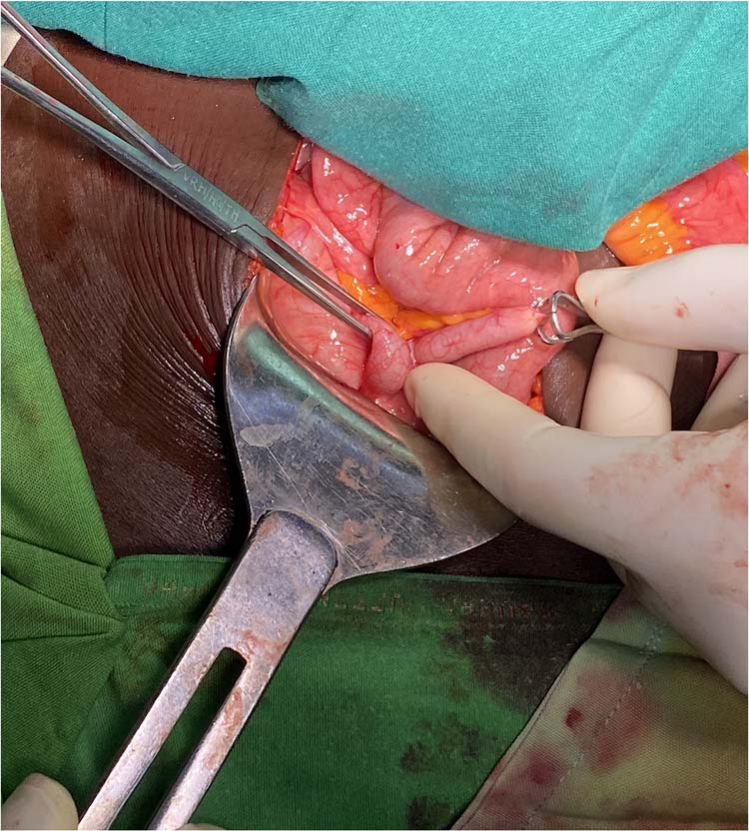
Inflamed appendix with adhesions angulating the distal part of it.

**Figure 5:**
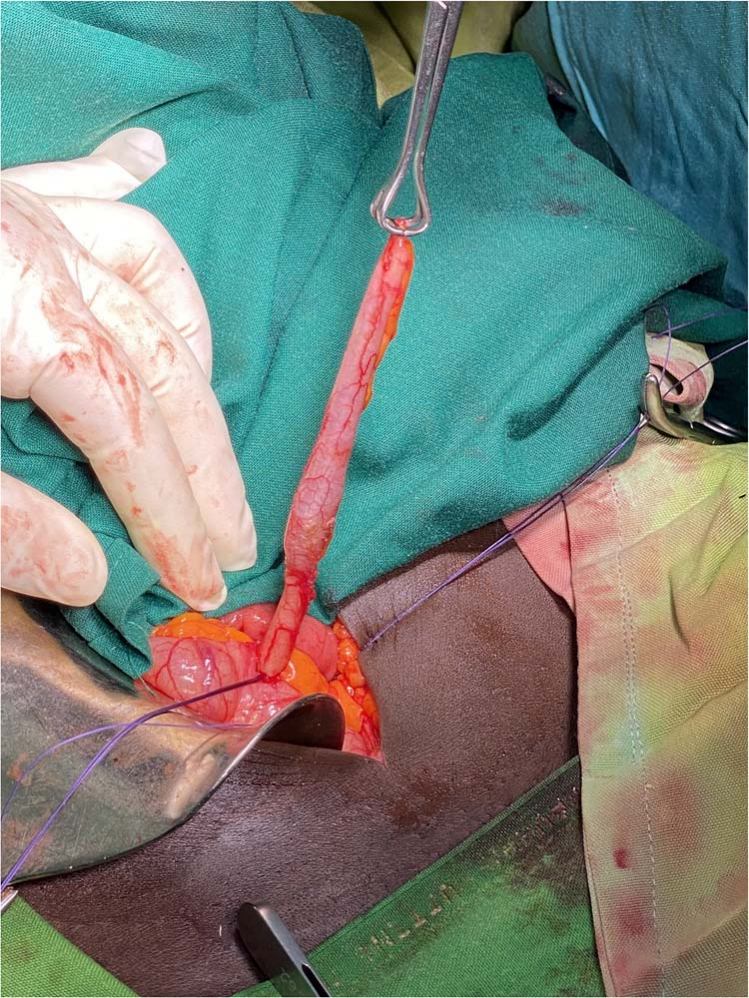
Inflamed and distended appendix from its distal third after being released from adhesions and mesoappendix.

Explorer laparotomy was done founding a descendent mesenteric colon cyst of about 10 cm in both diameter, which was fully enucleated conserving the left colic artery that was running over the anterior surface of the cyst (Fig. 3), and during the exploration of the abdomen, we found an inflamed appendix with increased vascularization and multiple adhesion of the base that make it to become angulated with a narrow base and dilated distal part (Figs 4 and 5). An appendectomy was done.

Histopathology reported a cyst measuring 11 × 9 × 8 cm, filled with 300 ml of dark brown fluid and lined on the wall by a simple epithelium without malignancy, concluding it as a simple mesenteric cyst. The appendix showed a dilated wall with a mild acute inflammatory infiltrate in the muscular layer and vascular congestion; there was the presence of an intestine fragment with lymphoid follicle proliferation and many lymphocytes in the mucosa, concluding it as an acute appendicitis.

The patient was discharged on post-operative Day 5 and was followed up in outpatient consultation for 10 months without complications or recurrence.

## DISCUSSION

A mesenteric cyst is one of the most uncommon abdominal tumours, with only about 820 cases reported since 1507. The incidence ranges from one per 100 000 to 250 000 admissions [6, 7]. Most authors describe a wide range of ages, from 22 to 46 years, but the prevalence is highest in the fourth decade of life [2]. However, cases have been documented during childhood, with a mean age of presentation of 2 years and a male-to-female ratio of 5:2 [8]. Our patient’s age at the time of diagnosis was 51.

Abdominal pain is documented as the most common symptom; rarely is an abdominal distention or a mass found on physical examination. About 10% of patients present with acute complications such as intestinal obstruction, volvulus, torsion, intra-cystic bleed and leakage of infective fluid [9]. Our patient maintained a history of intermittent stomach pain with a period of exacerbation that, in our judgement, was associated with the acute inflammation of the vermiform appendix.

Most authors (50–67%) reported that the most common location for mesenteric cysts is the mesentery of the small intestine; ~24–37% of cysts were discovered in the mesocolon, and <1% of the cases have been reported in the mesentery of the descending colon, sigmoid or rectum [5]. Another 14.5% of cysts were found in the retroperitoneum [2]. A left mesocolon cyst was revealed in our patient.

Imaging diagnostics of the mesenteric cyst are based on abdominal ultrasound, CT and magnetic resonance imaging [8, 10]. In our case, the CT scan once again proved its worth, as it confirmed the presence of the cyst and acute appendicitis. Because of its high diagnostic accuracy in identifying signs of appendiceal thickening and inflammation, CT remains the imaging modality of choice in evaluating adult patients with acute appendicitis [11]; its rate of accuracy is between 93 and 97% [12]. Ultrasonography, which is a valuable first-pass modality for appendix evaluation, [13] was ineffective in our case possibly because of its reliance on the operator.

Standard therapy for mesenteric cysts is total resection. This procedure could be performed laparoscopically or via laparotomy [3]. Recurrence and morbidity have been linked to partial excision [14]. The post-operative recurrence rate is low, ranging from 0 to 13.6% [5]. If possible, enucleation can be performed without removing the bowel, as we did in our case without any complications. Nevertheless, if the cyst cannot be safely separated from the bowel, it is necessary to remove the bowel along with the cyst and complete the surgery by anastomosing the cut bowel ends [1]. Simple aspiration and marsupialization are not recommended because both have an unacceptable high rate of recurrence and infection [2].

## CONCLUSION

Despite a rise in the publication of case reports, mesentery cysts are generally an uncommon and poorly studied disease. Particularly in adult patients, their association or presentation with acute appendicitis makes them even rarer, necessitating clinical expertise and radiological images for a conclusive diagnosis. This case report could be a good starting point for other type of research that help to understand better the association between different intrabdominal conditions.

## FUNDING

I acknowledge no financial support from any institutions or organizations.

## CONFLICT OF INTEREST STATEMENT

None declared.
